# Participant-Reported Reductions in Falls and Fall-Related Injuries Associated With Variable-Friction Footwear: Retrospective Pre-Post Survey Study

**DOI:** 10.2196/104177

**Published:** 2026-09-22

**Authors:** Sara Nataletti, Jennifer Bartloff, Sara Prokup, Joseph Harris, Anushka Larson, Tyler Susko, Arun Jayaraman

**Affiliations:** 1 Max Näder Center for Rehabilitation Technologies and Outcomes Research, Shirley Ryan AbilityLab, 355 E. Erie St, Chicago, IL, 60611, United States, 1 312 238 6875; 2 Department of Physical Medicine & Rehabilitation, Feinberg School of Medicine, Northwestern University, 710 N. Lake Shore Dr, Chicago, IL, United States; 3 Technology and Innovation Hub, Shirley Ryan AbilityLab, Chicago, IL, United States; 4 Cadense, Goleta, CA, United States; 5 Department of Mechanical Engineering, University of California, Santa Barbara, Santa Barbara, CA, United States; 6 Department of Physical Therapy & Human Movement Sciences, Feinberg School of Medicine, Northwestern University, Chicago, IL, United States

**Keywords:** falls, fall prevention, rehabilitation, mobility impairment, assistive devices

## Abstract

**Background:**

Falls represent a critical public health burden in older adults and individuals with neurological disabilities, but evidence for passive footwear-based fall prevention strategies remains limited.

**Objective:**

This study aimed to evaluate changes in fall frequency and fall-related outcomes following adoption of variable-friction (VF) footwear.

**Methods:**

This retrospective pre-post observational study used self-reported survey data collected from community-dwelling adults aged 18 years or older who had worn VF footwear for 3 months or longer. Participants were recruited from a convenience sample of 14,570 invited customers who purchased the device through an electronic survey platform. Outcomes included self-reported fall frequency, injurious falls, fall mechanisms, fall-related health care use (including medical visits, urgent care, and hospitalization), and assistive device use, assessed before and during VF footwear use.

**Results:**

A total of 706 participants completed the survey, yielding 705 participants with usable data for analysis (4.8% response rate). Mean monthly participant-reported falls decreased from 2.4 (SD 4.3) before VF use to 0.3 (SD 1.9) during VF use (87.1% reduction; Wilcoxon signed-rank test, *P*<.001). The proportion reporting zero falls increased from 22.7% to 84.4%. Among participants with at least 1 fall before VF use (n=545), 95.4% (n=520) reported a reduction, including 80.7% (n=440) reporting zero falls during VF use. Injurious falls decreased by 93.2% (*P*<.001). Participants requiring medical attention decreased from 192 (27.2%) to 12 (1.7%; 93.8% reduction; *P*<.001), and hospital or urgent care visits decreased from 131 (18.6%) to 10 (1.4%; 92.4% reduction; *P*<.001). Trip-related falls decreased from 52.3% to 31.5% of reported mechanisms among participants who reported a fall mechanism in both periods (*P*<.001). Assistive device use decreased from 70.8% to 16.6% (*P*<.001). Mean annualized insurance-related fall costs decreased from US $5946.2 to US $45.7 per person-year (99.2% reduction) across the full cohort.

**Conclusions:**

VF footwear use was associated with substantial reductions in self-reported falls, injurious falls, and fall-related health care use. Reductions in trip-related falls are consistent with the proposed biomechanical mechanism of the device. Given the low survey response rate and potential for self-selection bias, these findings should be considered preliminary. Prospective controlled studies are needed to confirm these findings.

## Introduction

Falls are among the most consequential and costly public health events affecting older adults [1-3]. Approximately 25% to 33% of adults aged 65 years and older experience at least one fall annually, with rates approaching 50% among those over 75 [4]. More than one-third of these falls result in injuries, including fractures and traumatic brain injuries that can precipitate functional decline, institutionalization, and increased mortality [5-8]. Health care spending attributable to nonfatal falls among older adults in the United States grew from approximately US $49.5 billion in 2015 to US $80 billion in 2020, with costs projected to exceed US $100 billion by 2030 [9,10]. These figures reflect direct medical expenditures alone and do not capture the broader costs of lost independence, caregiver burden, and reduced quality of life.

Beyond physical injury, falls contribute to fear of falling [11,12], affecting an estimated 21% to 85% of older adults [13,14], which itself doubles future fall risk and creates a self-reinforcing cycle of declining activity and increasing vulnerability [15-17]. This heightened vulnerability also reflects age-related declines in postural stability. Progressive deterioration in balance control, particularly under conditions of reduced sensory input, impairs the ability to maintain stability and recover from balance disturbances, further increasing susceptibility to falls in older adults [18]. This burden is further compounded in individuals with neurological conditions such as multiple sclerosis, stroke, and Parkinson disease, where disability-related mobility impairments add to the already elevated risk associated with aging [19-23]. In a national sample of adults with multiple sclerosis aged 55 to 94 years, 64% reported at least 2 falls per year, and more than half experienced injurious falls [24].

Current evidence-based strategies for fall prevention include multicomponent exercise and balance training programs, which can reduce fall rates by 15% to 30% [25-30]. However, their population-level impact remains constrained by poor long-term adherence, with fewer than 1 in 4 participants continuing prescribed programs at 12 months, and supervised in-person programs face persistent barriers of cost, travel, and accessibility [31-34]. Footwear represents a complementary strategy of a fundamentally different kind: worn daily as part of normal routine, it continuously modifies the mechanical interface between the person and their environment without requiring any behavioral commitment. Prior work has established that design features including low heel height, slip-resistant outsoles, proper fit, and secure fastening are associated with improved stability and reduced fall risk [35-37]. Beyond these structural features, perceived footwear comfort and mediolateral support have also been associated with fear of falling and perceived stability in older adults, suggesting that both the physical design and user perception of footwear may influence factors related to fall risk [38]. Rocker-sole and balance-training shoes have also been investigated to enhance neuromuscular control, though evidence remains mixed [39,40].

Among the most common precipitating causes of falls are forward trips, occurring when toe clearance fails during the swing phase, producing abrupt foot deceleration while the center of mass continues forward [41,42]. Variable-friction (VF) footwear has been proposed as a novel strategy to mitigate this mechanism by allowing controlled sliding of the toe during a failed clearance event, thereby reducing the abrupt stopping force that contributes to forward falls. Cadense Original shoes (Cadense Inc) incorporate this system, offering a targeted, mechanism-specific, and adherence-free approach to fall prevention during normal walking. However, evidence evaluating friction-modifying footwear as a real-world fall prevention strategy is limited.

Evaluating such interventions at scale requires reliable yet feasible methods of falls ascertainment. While prospective falls calendars are considered the gold standard, their logistical burden limits applicability in large community-based studies [43,44]. Self-reported surveys offer a practical and well-validated alternative, with prior studies demonstrating accurate recall of falls over 12 months, particularly for injurious falls, and good agreement with prospective methods [44-46].

This study examined changes in fall frequency and fall-related consequences following the adoption of VF footwear, assessed via self-reported survey. Outcomes included fall frequency, injurious falls, fall mechanisms, and fall-related health care use. We hypothesized that participants wearing VF shoes would experience a reduction in fall prevalence, driven primarily by fewer forward trip-related events.

## Methods

### Study Design

This was a retrospective pre-post observational study using self-reported survey data to evaluate fall frequency and fall-related outcomes before and during the use of a VF footwear device. Exposure and outcomes were assessed through a single survey administration, with participants retrospectively reporting outcomes for 2 periods: before and during VF footwear use. The study was conducted anonymously via an electronic survey platform (REDCap). This study was reported in accordance with the STROBE (Strengthening the Reporting of Observational Studies in Epidemiology) guidelines (Checklist 1).

### Participants

VF footwear was not provided or administered as part of this study. Rather, participants were existing Cadense customers who had independently purchased and worn the Cadense Original VF shoe for at least 3 months before survey completion. Cadense identified customers who met this minimum use criterion and provided the corresponding contact information to the research team at Shirley Ryan AbilityLab, thereby establishing the pool of potentially eligible participants. All recruitment invitations were subsequently distributed directly by the Shirley Ryan AbilityLab team. Eligible participants were adults aged 18 years or older and able to complete the survey in English. Individuals with severe cognitive or communication deficits or who were prisoners were excluded. Eligibility criteria were described in the recruitment email, and eligibility was self-evaluated by respondents prior to survey initiation. The survey link was distributed via email to all customers who met inclusion criteria. A total of 14,570 eligible customers were invited to participate in the survey. Recipients were given a response window of up to 4 weeks to complete the survey following receipt of the recruitment email.

### VF Footwear

**Figure 1.**
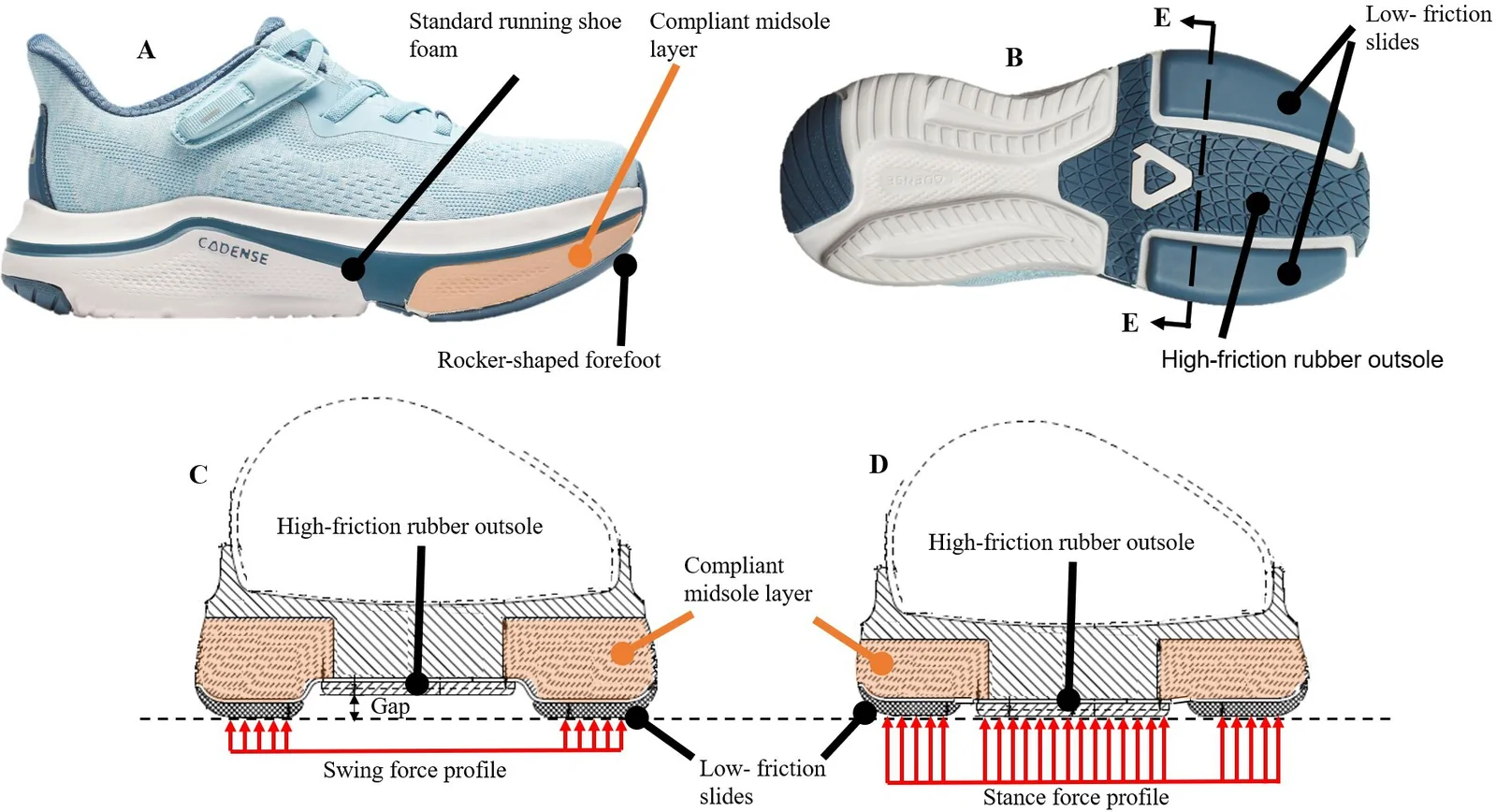
Variable-friction (VF) footwear design and functional concept. (A) Side view of the VF shoe showing the rocker-shaped forefoot intended to facilitate toe clearance during gait, the midsole made from standard running shoe foam, and the compliant midsole layer, which is critical to the functional concept. (B) VF outsole design showing the high-friction rubber outsole positioned between low-friction slides. (C) Section view through E-E illustrating the low-friction state seen during inadvertent swing-phase toe contact, where a small gap ensures contact force with only the low-friction slides, reducing orthogonal frictional forces that cause trips. (D) Section view through E-E illustrating the high-friction state during weight bearing, where compression of the compliant midsole layer extends the force profile to the high-friction rubber outsole, promoting high-grip stance and push-off.

The Cadense Original VF shoe (Figure 1) is a US Food and Drug Administration–registered Class I medical device. The device operates passively and alters frictional properties of the outsole based on loading conditions during gait. During the swing phase or low ground reaction force conditions, the shoe provides reduced friction at the forefoot region through low-friction polymer slides positioned slightly proud of the primary high-friction outsole (see the gap in Figure 1C), allowing controlled sliding during incidental toe contact. During stance, increased loading compresses a compliant midsole layer set beneath the low-friction slides, retracting the low-friction slides and bringing the high-friction rubber outsole into contact with the walking surface (see Figure 1D) to support stability during weight acceptance and push-off. The design incorporates a rocker-shaped forefoot intended to reduce toe scuff during gait much like a ski tip sliding over small imperfections in the walking surface. The transition between low- and high-friction states occurs automatically based on normal force during gait and does not require active components. The device contains no electronics or batteries and is intended to mitigate forward trip-related falls by modifying toe-ground interaction mechanics. The device also provides auditory feedback during toe contact events over hard surfaces, which may increase user awareness of foot clearance during walking.

### Data Collection

Data were collected using a custom electronic survey administered through REDCap, a secure web-based data management platform hosted by Shirley Ryan AbilityLab. This was a closed survey, accessible only via a unique link distributed directly to identified eligible customers; it was not publicly posted or advertised. The survey instrument was reviewed internally by the research team for usability and technical functionality prior to distribution. The survey was distributed via a unique REDCap link included in the recruitment email. The survey link led respondents through 2 screens: an initial screen presenting the consent form, with an option to download a PDF copy, followed by a second screen presenting the full survey (Multimedia Appendix 1). The survey comprised 10 primary items, several with conditionally displayed follow-up questions based on branching logic; items were presented in a fixed order and were not randomized, consistent with this branching logic. Survey responses were considered complete only after respondents reached the final page and submitted the survey. Partial responses were not retained, and respondents who did not complete submission needed to restart the survey. Once a survey had been completed and submitted, REDCap’s built-in mechanism prevented the same respondent from submitting the survey more than once. The survey (Multimedia Appendix 1) was designed to characterize fall frequency and fall-related outcomes before and after use of the Cadense VF shoe. The instrument collected demographic information and clinical characteristics, including age, body weight, underlying medical condition associated with fall risk, and assistive device (AD) use, as well as retrospective information on reported falls during the year prior to initiating VF shoe use and during sustained use of the VF shoe (≥3 mo). Specifically, participants reported fall frequency, fall mechanism (eg, trip-related vs balance-related), occurrence of injurious falls, need for medical attention, health care use setting, and estimated out-of-pocket and insurance-related costs associated with fall events. Cost estimates were self-reported by participants as open numeric responses and were not verified against medical records or insurance claims. All questions were structured in closed-ended categorical or numeric formats to facilitate completion and minimize missing data. Skip logic was incorporated to display follow-up questions only when applicable (eg, injury-related items presented only to respondents reporting a fall). The survey required approximately 10 minutes to complete and was completed anonymously. Identifiable information provided for optional raffle entry was collected separately and was not linked to survey responses.

### Outcome Measures

The primary outcome of this study was the change in self-reported fall frequency before VF shoe use and during sustained VF shoe use (≥3 mo). The pre-VF reporting period covered the 12 months preceding VF shoe use, whereas the VF-use reporting period reflected each participant’s duration of VF shoe use and therefore varied across participants, with a minimum of 3 months. To account for these differing reporting periods, fall frequency was assessed as participants’ self-reported average number of falls per month during each period. Fall frequency was analyzed both as a continuous variable and as categorical outcomes, including the proportion of participants reporting at least one fall and those reporting zero falls.

Secondary outcomes included injurious falls and other fall-related consequences and characteristics. Given the lower frequency of injurious falls relative to total falls, the number of injurious falls and their consequences were reported over the respective reporting periods, rather than as a monthly rate. Fall-related health care use included whether participants required medical attention following a fall and whether care occurred in a hospital or urgent care setting. Economic outcomes included self-reported out-of-pocket and insurance-related costs associated with fall events.

Fall mechanisms were also collected and categorized as trip-related or nontrip-related events to examine potential changes in fall etiology. AD use was also evaluated as a mobility-related outcome. Participants reported use of ADs before and during VF shoe use, including canes, walkers, ankle-foot orthoses, and neuromuscular stimulation devices. Changes in overall AD use and device-specific use were examined.

Additional descriptive outcomes included the proportion of participants reporting fewer falls, no change, or increased falls during VF shoe use.

### Statistical Analysis

Descriptive statistics were used to characterize the study sample, including demographic variables and medical conditions associated with fall risk. Continuous outcomes are presented as both mean (SD) and median (IQR). Because fall frequency, injurious falls, and cost variables demonstrated nonnormal distributions, within-participant comparisons of continuous outcomes between the pre-VF and VF periods were performed using Wilcoxon signed-rank tests. Paired categorical outcomes were compared using McNemar tests, as appropriate. Effect sizes were reported as the matched-pairs rank-biserial correlation and mean difference with 95% CI (t-distribution-based) for Wilcoxon-tested outcomes, and as risk differences with 95% CI (Wald method) for McNemar-tested outcomes.

Changes in paired categorical outcomes, including fall occurrence, injurious falls, health care use, fall mechanisms, and AD use, were evaluated using McNemar tests or related paired chi-square procedures, as appropriate. A consistent paired analytic framework was applied across outcome domains to reflect the repeated-measures structure of the data.

Because the pre-VF reporting period was fixed at 12 months, whereas the VF-use reporting period varied across participants, differences in observation periods were addressed according to the method of outcome collection. Fall frequency, the primary outcome, was reported as an average monthly rate for both periods and therefore required no adjustment for exposure duration. In contrast, injurious falls and fall-related costs (out-of-pocket and insurance) were reported as totals over the respective reporting periods. To account for the variable VF-use observation window, during-VF totals were annualized for each participant by dividing the reported value by their individual VF-use duration in years before comparison with the corresponding 12-month pre-VF value. If a respondent indicated no injurious falls during a period, the out-of-pocket and insurance cost values were assigned a value of zero, consistent with the survey skip logic (Multimedia Appendix 1). Otherwise, missing cost values were retained as missing and excluded from paired analyses.

Health care use outcomes (need for medical attention, hospital or urgent care use) and the proportion of participants reporting at least one injurious fall were based on whether each event occurred during the respective reporting period. Unlike the corresponding continuous outcomes, these measures cannot be meaningfully annualized; these were therefore compared as raw proportions over the unequal exposure windows described above.

Fall mechanisms were analyzed among participants who reported mechanism information in both the pre-VF and VF-use periods. Paired changes in the occurrence of trip-related falls were evaluated using a McNemar-type test based on discordant pairs.

All analyses were performed using Python (version 3.11). Statistical significance was set at α=.05 (2-tailed).

### Ethical Considerations

Ethics approval for this study was granted by the Institutional Review Board of Northwestern University (STU00224455). Participants provided passive consent by reviewing an electronic consent form and proceeding to the survey, under an institutional review board–approved waiver of documentation of informed consent given the anonymous, minimal-risk nature of the study. The study procedures adhered to the principles of the Declaration of Helsinki and institutional ethical guidelines. All data collected were deidentified prior to analysis to protect participant privacy and confidentiality. At the conclusion of the study, all participants who completed the survey were entered into a random drawing to receive 1 of 5 free pairs of Cadense VF shoes (retail value US $199 each).

## Results

### Participant Characteristics

A total of 14,570 eligible customers were invited to participate. Of these, 706 (4.8%) completed the survey. One response was excluded due to a typographical data entry error in fall frequency, resulting in a final analytic sample of 705 participants. Demographic and clinical characteristics are summarized in Table 1.

**Table 1.** Participant characteristics (N=705).

Characteristic	Value
Age (y), mean (SD)	59.5 (13.8)
Weight (kg), mean (SD)	81.5 (20.4)
Duration of VF^a^ shoe use (mo), mean (SD)	12.3 (8.4)
Shoe style, n (%)	
Men’s	274 (38.9)
Women’s	431 (61.1)
Reported medical condition^b^, n (%)	
Multiple sclerosis	391 (55.5)
Peripheral neuropathy	127 (18.0)
Stroke	49 (7.0)
Spinal cord injury	42 (6.0)
Age-related condition	68 (9.6)
Traumatic brain injury	38 (5.4)
Parkinson disease	29 (4.1)
Charcot-Marie-Tooth disease	14 (2.0)
Other condition	131 (18.6)

a VF: variable-friction.

b Participants were allowed to select multiple conditions; therefore, percentages do not sum to 100%. Overall, 148 (21%) participants reported more than one condition.

### Fall Frequency

The median self-reported monthly falls per participant decreased from 1.0 (IQR 0.5‐3.0) prior to VF shoe use to 0.0 (IQR 0.0‐0.0) during VF shoe use (Wilcoxon signed-rank *P*<.001). The corresponding mean decreased from 2.4 (SD 4.3) to 0.3 (SD 1.9), representing an absolute reduction of 2.1 falls per month and an 87.1% relative reduction (Table 2). Overall, 73.8% (520/705) of participants reported a reduction in fall frequency, 24.5% (173/705) reported no change, and 1.7% (12/705) reported an increase. Reductions in participant-reported falls were observed across all major condition groups (see Table 3). The proportion of participants reporting zero falls increased from 22.7% (160/705) prior to VF use to 84.4% (595/705) during VF use. Among participants who reported at least one fall prior to VF shoe use (n=545), 95.4% (n=520) reported a reduction in falls, including 80.7% (n=440) who reported zero falls during VF use, and 1.3% (n=7) reported an increase in falls.

A small subgroup of participants (n=12) reported increased fall frequency during VF shoe use, with median (IQR) monthly falls increasing from 0.7 (IQR 0.0‐1.2) to 1.5 (IQR 1.0‐3.0). The corresponding mean increased from 2.1 (SD 4.3) to 3.8 (SD 5.7). Despite this increase, injurious falls did not worsen for most individuals: only 1 participant reported an increase in injurious falls, while 3 reported fewer and 8 remained unchanged. Examination of fall mechanisms showed a shift from primarily trip-related falls prior to VF use (5/9 with available data) to predominantly balance-related falls during VF use (10/12), including both loss of balance while walking and standing. This pattern suggests that increases in falls within this small subgroup were more likely related to underlying balance impairments rather than trip-related mechanisms targeted by the VF shoes.

**Table 2.** Outcomes^a^.

Outcome	Pre-VF^b^ shoe	Post-VF shoe	Change (%)	*P* value	Effect size, *r*	Mean Δ (95% CI)	RD^c^ percentage points (95% CI)
Falls/month, mean (SD)/median (IQR)	2.4 (4.3)/1.0 (0.5‐3.0)	0.3 (1.9)/0.0 (0.0‐0.0)	−87.1	<.001^d^	−0.97	−2.1 (−2.4 to −1.8)	—^e^
People with ≥1 fall, n (%)	545 (77.3)	110 (15.6)	−79.8	<.001^f^	—	—	−61.7 (−65.4 to −58.0)
People with 0 falls, n (%)	160 (22.7)	595 (84.4)	271.9	<.001^f^	—	—	+61.7 (+58.0 to +65.4)
Injurious falls/year, mean (SD)/median (IQR)	1.8 (4.2)/1.0 (0.0‐2.0)	0.1 (0.7)/0.0 (0.0‐0.0)	−93.2	<.001^d^	−0.95	−1.7 (−2.0 to −1.4)	—
People with ≥1 injurious fall, n (%)	381 (54.0)	43 (6.1)	−88.7	<.001^f^	—	—	−47.9 (−51.7 to −44.2)
People requiring medical attention, n (%)	192 (27.2)	12 (1.7)	−93.8	<.001^f^	—	—	−25.5 (−28.8 to −22.3)
Participants requiring hospital or urgent care, n (%)	131 (18.6)	10 (1.4)	−92.4	<.001^f^	—	—	−17.2 (−20.0 to −14.3)
Out-of-pocket fall cost, $/person-year, mean (SD)/median (IQR)	281.2 (2069.8)/0.0 (0.0‐0.0)	17.2 (266.8)/0.0 (0.0‐0.0)	−93.9	<.001^d^	−0.91	−264.1 (−417.7 to −110.5)	—
Insurance fall cost, $/person-year, mean (SD)/median (IQR)	5946.2 (94,754.1)/0.0 (0.0‐0.0)	45.7 (505.0)/0.0 (0.0‐0.0)	−99.2	<.001^d^	−0.95	−5900.6 (−12,906.5 to 1105.4)	—
People using assistive device, n (%)	499 (70.8)	117 (16.6)	−76.6	<.001^f^	—	—	−54.0 (−57.9 to −50.2)

a Continuous outcomes are presented as mean (SD) and median (IQR). Percentages based on the 705 sample size. Changes are calculated from the mean values as % changes [(post−pre)/pre] × 100.

b VF: variable-friction.

c RD: risk difference.

d Wilcoxon signed-rank test; *r*: matched-pairs rank-biserial correlation.

e Not applicable.

f McNemar test.

**Table 3.** Proportion of participants reporting reductions in falls and injurious falls by medical condition.

Condition	Participants (N)	Fewer falls, n (%)	Fewer injurious falls, n (%)
Multiple sclerosis	391	305 (78)	219 (56)
Peripheral neuropathy	127	96 (75.6)	76 (59.8)
Age-related conditions	68	49 (72.1)	32 (47.1)
Parkinson disease	29	18 (62.1)	15 (51.7)
Spinal cord injury	42	28 (66.7)	23 (54.8)
Traumatic brain injury	38	25 (65.8)	16 (42.1)
Stroke	49	32 (65.3)	20 (40.8)

### Fall Mechanism

Trip-related events accounted for 67.7% (397/586) of reported fall mechanisms prior to VF shoe use, followed by loss of balance while walking (138/586, 23.5%) and loss of balance while standing (51/586, 8.7%). During VF shoe use, trip-related events accounted for 30.7% (42/137) of reported fall mechanisms, while loss of balance while walking became the most frequently reported mechanism (61/137, 44.5%), followed by loss of balance while standing (34/137, 24.8%). Of the 586 reported fall mechanisms prior to VF use, 130 also were reported a mechanism during VF use. To provide a paired within-participant comparison, we examined fall mechanisms among these 130 participants who had reported a mechanism in both periods. In the period before VF use, trip-related events accounted for 52.3% (68/130) of reported mechanisms in this subset, followed by loss of balance while walking (44/130, 33.8%) and loss of balance while standing (18/130, 13.8%). During VF use, trip-related events decreased to 31.5% (41/130), while loss of balance while walking became the most frequently reported mechanism (55/130, 42.3%), followed by loss of balance while standing (34/130, 26.2%). This paired reduction in trip-related falls was statistically significant (McNemar-type test on discordant pairs, *P*<.001). These findings are consistent with a shift in the predominant mechanism of falls from primarily trip-related events before VF use to primarily balance-related mechanisms during VF use.

### Injurious Falls and Medical Attention

The median annual self-reported injurious falls decreased from 1.0 (IQR 0.0‐2.0) prior to VF shoe use to 0.0 (IQR 0.0‐0.0) during VF use (Wilcoxon signed-rank test, *P*<.001). The corresponding mean decreased from 1.8 (SD 4.2) to 0.1 (SD 0.7), representing a 93.2% reduction. Consistent with this reduction, 52.6% (371/705) of participants reported fewer injurious falls, 46.5% (328/705) reported no change, and 0.9% (6/705) reported an increase. The number of participants reporting at least one injurious fall per reporting period decreased from 54% (381/705) prior to VF shoe use to 6.1% (43/705) during VF use (McNemar test, *P*<.001). The number of participants requiring medical attention for falls decreased from 27.2% (192/705) prior to VF shoe use to 1.7% (12/705) during VF use, representing a 93.8% reduction (McNemar test, *P*<.001). Similarly, hospital or urgent care visits decreased from 18.6% (131/705) to 1.4% (10/705), corresponding to a 92.4% reduction (McNemar test, *P*<.001).

### Fall-Related Costs

Annualized mean out-of-pocket fall-related costs decreased from $281.2 (SD $2069.8) per person-year prior to VF shoe use to $17.2 (SD $266.8) per person-year during VF shoe use, a 93.9% reduction (Wilcoxon signed-rank test, *P*<.001). Median out-of-pocket costs were $0 (IQR $0-$0) in both periods, reflecting that the majority of participants reported no fall-related out-of-pocket cost in either period. Before VF shoe use, 21.8% (154/705) of participants reported a nonzero cost, compared with 1.6% (11/705) during VF shoe use.

Similarly, annualized mean insurance-related fall costs decreased from $5946.2 (SD $94,754.1) per person-year to $45.7 (SD $505.0) per person-year, a 99.2% reduction (Wilcoxon signed-rank test, *P*<.001). As with out-of-pocket costs, median insurance costs were $0 (IQR $0-$0) in both periods. Before VF shoe use, 24.3% (171/705) of participants reported a nonzero insurance cost, versus 1.4% (10/705) during VF shoe use.

### AD Use

The proportion of participants reporting use of any AD decreased from 70.8% (499/705) prior to VF shoe use to 16.6% (117/705) during VF use (McNemar test, *P*<.001). Overall, 55.2% (389/705) of participants discontinued AD use while using VF shoes, whereas only 1.0% (7/705) initiated AD use. Similar reductions were observed across individual device categories, including walker use (34.4% to 8.9%), cane use (49.7% to 11.8%), ankle-foot orthosis use (29.2% to 3.0%), and neuromuscular electrical stimulation device use (12.7% to 3.0%), with all changes significant based on McNemar tests for paired proportions (all *P*<.001).

## Discussion

### Principal Findings

The primary objective of this study was to evaluate the association between the use of VF footwear and participant-reported changes in fall frequency, fall-related injuries, and health care use. These findings should be interpreted in the context of the low survey response rate (706/14,570, 4.8%) and the requirement for participants to have used VF footwear for at least 3 months, as respondents may not be representative of the broader population of VF footwear users. Individuals who perceived greater benefit or were able to continue using the footwear may have been more likely to be represented in the study. Among respondents, substantial and statistically significant reductions were reported across all primary and secondary outcomes following the adoption of VF footwear. During VF footwear use, participants reported an 87.1% reduction in mean monthly fall frequency (2.4 to 0.3 falls/month) and a 93.2% reduction in injurious falls (1.8 to 0.1 falls/year). Notably, 95.4% (520/545) of participants who reported at least one fall in the previous year also reported a reduction in fall frequency during VF shoe use. Although these findings require confirmation in prospective controlled studies, they are particularly relevant given the study population, which comprised individuals with significant neurological and age-related conditions, such as multiple sclerosis, neuropathy, stroke, and Parkinson disease, who are historically at a higher risk for falls [20].

The magnitude of the reported reduction exceeds that typically observed in randomized trials of exercise and multifactorial fall-prevention interventions. A recent meta-analysis of 108 randomized controlled trials involving 23,407 participants reported an average 23% reduction in fall rates following exercise interventions [47]. Similarly, systematic reviews of multifactorial interventions have reported reductions of approximately 23% to 24% [48,49]. Although direct comparison with the present findings is limited by differences in participant populations, baseline fall risk, outcome ascertainment, follow-up duration, and study design, several factors may therefore explain the larger associations observed in the present study. Participants self-reported neurological and age-related conditions associated with high risk of recurrent falls, potentially allowing greater opportunity for improvement than participants enrolled in many previous fall-prevention trials. Furthermore, VF footwear is designed to address a specific mechanical contributor to trip-related falls, whereas most established interventions primarily target strength, balance, or physical function. At the same time, the retrospective observational design, absence of a concurrent control group, and the potential influence of selection bias, nonresponse bias, recall bias, expectation bias, regression to the mean, and uncontrolled changes in health status or treatment may also have contributed to the observed effect size. Self-selection may be particularly relevant when interpreting the magnitude of these reductions, as customers who perceived greater benefit may have been more likely to participate than those with less favorable experiences.

A key finding of this study is the significant shift in reported fall mechanisms. Prior to using the VF footwear, for participants reporting trip-related events before and after VF use, 52.3% (68/130) of all falls were due to trips, a figure consistent with existing literature identifying forward trips as a leading cause of balance loss in older adults and those with physical disabilities [50,51]. During VF footwear use, for these users, the reported trip-related falls dropped to 31.5% (41/130). This finding is particularly relevant within the broader context of fall prevention, where age-related declines in postural stability and neurological impairments increase vulnerability to balance disturbances and make recovery from trip-related events more challenging. Previous studies have also reported that footwear comfort and perceived mediolateral stability are associated with fear of falling and perceived stability in older adults, further highlighting the potential role of footwear as one component of comprehensive fall prevention strategies [18,38]. Although the present study did not directly evaluate postural control or footwear comfort, the observed reduction in trip-related falls is consistent with the proposed biomechanical mechanism of VF footwear, whereby low-friction elements at the toe allow controlled sliding during incidental toe-ground contact rather than the abrupt deceleration that precipitates forward falls.

Reported reductions were observed consistently across diagnostic groups. Participants with multiple sclerosis reported the largest improvements, with 78% (305/391) reporting fewer falls and 56% (219/391) reporting fewer injurious falls. Similar patterns were observed in peripheral neuropathy, Parkinson disease, spinal cord injury, traumatic brain injury, stroke, and age-related conditions. Although these subgroup findings are descriptive and should not be interpreted as evidence of differential treatment effects, the consistency of the reported associations across clinically heterogeneous populations supports further prospective investigation of VF footwear in individuals at elevated risk of falling.

Similarly, self-reported fall-related health care use decreased substantially. Participants requiring medical attention after a fall decreased by 93.8% (192 to 12 participants), and reported hospital or urgent care visits decreased by 92.4% (131 to 10 participants). Although these reductions were larger than those reported in previous randomized trials, the direction of the findings is consistent with evidence showing that effective fall-prevention interventions can reduce subsequent health care use. For example, the GAPcare randomized controlled trial demonstrated fewer subsequent emergency department visits following an emergency department–initiated multidisciplinary intervention [52,53]. Given the well-established association between falls, downstream disability [10], and the substantial health care and economic burden attributable to falls [9,54], the observed participant-reported reductions in falls may have important clinical and economic implications if confirmed in prospective controlled studies. Because health care use and associated costs were self-reported and not independently verified through medical records, insurance claims, or other objective sources, these findings should be interpreted cautiously as preliminary estimates of potential impact rather than evidence of confirmed reductions attributable to VF footwear.

Participants also reported substantially reduced use of AD. Discontinuation of walkers, canes, and ankle-foot orthoses may reflect greater perceived stability, confidence, and functional independence. Given the known relationship between fear of falling, activity restriction, and functional decline, interventions that improve perceived safety during ambulation may have broader implications for participation and independence [11,55]. Previous randomized controlled trials have shown that reducing fear of falling can decrease activity restriction and improve confidence among older adults [56]. However, because balance, walking performance, or the clinical appropriateness of AD discontinuation were not objectively assessed, reduced AD use should not be interpreted as evidence of improved independence or as a recommendation to discontinue prescribed devices. Future prospective studies should evaluate changes in device use together with objective mobility outcomes and clinician assessment.

### Study Limitations

Several limitations must be considered when interpreting these results. First, this was a retrospective pre-post observational study without a concurrent control group; therefore, the results demonstrate an association between VF footwear use and reported outcomes but do not establish causality. Changes in falls may have been influenced by unmeasured confounders, including rehabilitation, medication changes, disease progression or recovery, environmental modifications, altered activity levels, or increased caution. Regression to the mean may also have contributed, as participants may have sought the footwear following a period of unusually frequent falls.

Second, the low response rate is a major limitation and restricts confidence that the respondents were representative of all invited customers. Individuals who perceived substantial benefit may have been more motivated to complete the survey, potentially inflating the observed association. Conversely, participants with less favorable experiences, who continued to fall, experienced discomfort or safety concerns, or were dissatisfied with the footwear may have been less likely to respond. Because outcomes among the large majority of invited customers who did not participate are unknown, the direction and magnitude of this potential nonresponse bias cannot be determined. The low response rate therefore limits generalizability and may have affected the observed relationships.

Third, eligibility required participants to have worn the footwear for at least 3 months. Although this ensured meaningful exposure, it may have excluded individuals who discontinued use because of discomfort, lack of perceived benefit, or other reasons, introducing survivorship bias. This may have preferentially selected individuals who tolerated and continued using the footwear, potentially contributing to an overestimation of the observed benefit. Future studies should prospectively enroll participants at the time the footwear is dispensed and document adherence, discontinuation, adverse experiences, and reasons for stopping use.

Fourth, eligibility criteria, participants’ characteristics, and all outcomes were self-reported and retrospectively recalled. Consequently, the findings are susceptible to misclassification, reporting, expectation, and recall bias. Because participants were aware of the intended purpose of VF footwear, their responses may have been influenced by expectations regarding its effectiveness. In addition, differences in the recall periods before and during VF footwear use may have affected the accuracy of reported fall frequencies. This was particularly relevant for binary outcomes, including the need for medical attention, hospital or urgent care use, and the occurrence of at least one injurious fall, which could not be meaningfully annualized and were therefore compared as raw proportions. The absence of objective outcome verification further limits the interpretation of these findings, as falls, injuries, diagnoses, and health care use were not confirmed through prospective monitoring, medical records, or insurance claims.

Finally, the survey collected only limited demographic and clinical information. Variables such as sex, comorbidities, disease severity, and functional status were not available, and the collected participant characteristics and diagnoses were based solely on self-report rather than independent clinical verification. Consequently, we were unable to evaluate whether the observed associations differed across important patient subgroups or account for demographic and clinical factors that may influence gait, balance, footwear perception, and fall risk. Previous work has shown that footwear comfort and foot structure may differ between women and men, particularly in older adults, suggesting that responses to footwear interventions may also vary [57]. Future prospective studies should include balanced recruitment and prespecified subgroup analyses to evaluate potential differences across demographic and clinical populations.

Despite these limitations, the consistency of the reported improvements across multiple outcomes and diagnostic groups supports further investigation. These findings should be considered preliminary real-world evidence that participants reported fewer falls and fall-related consequences during VF footwear use.

### Conclusions

In this retrospective survey, participants reported fewer falls, fewer injurious falls, and lower fall-related health care use while using VF footwear. Similar improvements were observed across multiple outcomes, including a shift away from trip-related falls. Although gait, balance, and footwear biomechanics were not directly evaluated, these findings are consistent with the proposed biomechanical rationale of the device. Likewise, the reported reduction in AD use may reflect greater perceived stability or confidence, but this was not directly assessed and should not be interpreted as evidence of improved functional mobility.

Given the study’s retrospective design, absence of a concurrent control group, and reliance on self-reported data, these findings should be considered preliminary associations rather than evidence of a causal treatment effect. Importantly, the low survey response rate raises substantial potential for nonresponse and self-selection bias. Participants who chose to respond may have differed systematically from the broader population of VF footwear users, including the possibility that those who experienced greater perceived benefit were more likely to participate. The magnitude of the observed associations may therefore not be representative of the full population of VF footwear users, limiting generalizability of these findings.

Prospective, controlled studies with objective outcome verification are needed to determine whether VF footwear causally reduces fall risk, to quantify its effect relative to existing fall-prevention interventions, and to identify subpopulations most likely to benefit; such studies should also collect a more comprehensive set of demographic and clinical covariates to enable multivariable analysis.

## Supplementary material

10.2196/104177 Multimedia Appendix 1 Survey contents.

10.2196/104177 Checklist 1 STROBE checklist.

## Abbreviations

AD: assistive device
STROBE: Strengthening the Reporting of Observational Studies in Epidemiology
VF: variable-friction
