# Supplementary material for: Participant-Reported Reductions in Falls and Fall-Related Injuries Associated With Variable-Friction Footwear: Retrospective Pre-Post Survey Study
Source: JMIR Rehabil Assist Technol. 2026 Sep 22;13:e104177. doi: 10.2196/104177 (PMC13596762; doi:10.2196/104177)
Supplement: Supplementary file 2 [file rehab-v13-e104177-s001.docx]

Survey Contents:

Thank you for taking the time to complete our fall-related research survey.

1. Please enter your age in years.
2. What style shoes do you wear? (Men’s/Women’s)
3. Please enter your estimated body weight in pounds.
4. Approximately, what was the date you began using the Cadense shoes?

**The following questions will ask you about falls prior to wearing your Cadense shoes**

5. Did you fall in the year prior to wearing your Cadense Original shoes?
   a. If yes:
      i. On average, how many falls did you have per month before you started wearing your Cadense Original shoes?
      ii. Check all changes in daily life that occurred due to these falls (Limited daily activities, Took time off from work, Experienced depression, Increased far of falling, Other_______)
          1. If Other selected:
             a. Since you selected ‘other’, please describe
      iii. If you fell in the year prior to wearing your Cadense Original shoes, how many falls caused an injury?
           1. If >0
              a. Check all injuries that apply (Cut, Bruise, Broken bone, Other _________)

i. If Other Selected:

1. Since you selected ‘other’, please describe

iv. If you fell in the year prior to wearing your Cadense Original shoes, how many falls required you to seek medical attention for the fall?
    1. If>0
       a. Where did you go to be seen (Primary Care Doctor, Hospital, Urgent Care, Physical therapist, Other ______)

i. If Other selected:

1. Since you selected ‘other’, please describe

b. Please estimate the out of pocket costs (for example, copays, equipment purchases, etc.) for your falls in the year before wearing your Cadense shoes ____________
c. Please estimate the cost to your insurance company for falls in the year before wearing your Cadense shoes _______
v. Before wearing your Cadense Original shoes, what were the main causes of your falls? (Trip and fall, Losing balance while standing, Losing balance while walking other than tripping).

6. What walking assistive devices did you use before getting your Cadense shoes (check as many as apply)? (Walker, Cane, AFO, Muscle stimulation device, Not Applicable, Other __________)
   a. If Other selected
      i. Since you selected ‘other’, please describe

**The following questions will ask you about falls while wearing your Cadense shoes**

7. Have you had any falls while wearing your Cadense Original Shoes?
   a. If yes:
      i. On average, how many falls did you have per month while wearing your Cadense Original Shoes?
      ii. Check all changes in daily life that occurred due to these falls (Limited daily activities, Took time off from work, Experienced depression, Increased fear of Falling, Other______)
          1. If Other selected:
             a. Since you selected ‘other’, please describe
      iii. If you fell while wearing your Cadense Original shoes, how many falls caused an injury?
           1. If >0
              a. Check all injuries that apply (Cut, Bruise, Broken bone, Other _________)

i. If Other selected

1. Since you selected ‘other’, please describe

iv. If you fell while wearing your Cadense Original shoes, how many falls required you to seek medical attention for the fall?
    1. If>0
       a. Where did you go to be seen (Primary Care Doctor, Hospital, Urgent Care, Physical therapist, Other ______)

i. If Other selected

1. Since you selected ‘other’, please describe

b. Please estimate the out of pocket costs for your falls while wearing your Cadense Original Shoes ____________
c. Please estimate the cost (for example, copays, equipment purchases, etc.) to your insurance company for falls while wearing your Cadense Original Shoes _______
v. While wearing your Cadense Original shoes, what were the main causes of your falls? (Trip and fall, Losing balance while standing, Losing balance while walking other than tripping).

8. What walking assistive devices do you use with your Cadense shoes (check as many as apply)? (Walker, Cane, AFO, Muscle stimulation device, Not Applicable, Other __________)
   a. If Other selected
      i. Since you selected ‘other’, please describe
9. In the time period after you started wearing your Cadense shoes, how many times did you fall per month when you were not wearing your Cadense shoes?

**The following question will ask you a little more about conditions that lead to falls.**

10. What is the condition that leads you to fall? (check all that apply) (Multiple Sclerosis, Stroke, Parkinson’s Disease, Neuropathy, Age, Traumatic Brain Injury, Spinal Cord injury, Myositis, Charcot-Marie-Tooth disease, Other _________
    a. If Other selected
       i. Since you selected ‘other’, please describe

Thank you for taking our survey. If you would like to be enrolled into a drawing for a free pair of Cadense Original shoes at the conclusion of this study, please click here to enter your name and contact information on the next form. Otherwise, please exit this browser window.
